# Prevalence and factors associated with frailty and pre-frailty in the older adults in China: a national cross-sectional study

**DOI:** 10.3389/fpubh.2023.1110648

**Published:** 2023-07-24

**Authors:** Xue-zhai Zeng, Ling-bing Meng, Ying-ying Li, Na Jia, Jing Shi, Chi Zhang, Xing Hu, Jia-bin Hu, Jian-yi Li, Di-shan Wu, Hui Li, Xin Qi, Hua Wang, Qiu-xia Zhang, Juan Li, De-ping Liu

**Affiliations:** ^1^Department of Cardiology, Beijing Hospital, National Center of Gerontology, Institute of Geriatric Medicine, Chinese Academy of Medical Sciences, Beijing, China; ^2^Department of Geriatrics, Beijing Hospital, National Center of Gerontology, Institute of Geriatric Medicine, Chinese Academy of Medical Sciences, Beijing, China; ^3^The Key Laboratory of Geriatrics, Beijing Institute of Geriatrics, Institute of Geriatric Medicine, Chinese Academy of Medical Sciences, Beijing Hospital/National Center of Gerontology of National Health Commission, Beijing, China; ^4^Health Service Department of the Guard Bureau of the Joint Staff Department, Beijing, China; ^5^China Research Center on Ageing, Beijing, China; ^6^Institute of Psychology, Chinese Academy of Sciences, Beijing, China

**Keywords:** China, older adults, frailty, pre-frailty, prevalence

## Abstract

**Objective:**

Frailty increases poor clinical outcomes in older adults, the aim of this study was to investigate the prevalence and factors associated with frailty and pre-frailty in older adults in China.

**Research design and methods:**

Data were obtained from the Sample Survey of the Aged Population in Urban and Rural China in 2015, which was a cross-sectional study involving a nationally representative sample of older adults aged 60 years or older from 31 provinces/autonomous regions/municipalities in mainland China. The frailty index (FI) based on 33 potential deficits was used to classify individuals as robust (FI < 0.12), pre-frail (FI ≧0.12 and <0.25) and frail (FI ≥0.25).

**Results:**

A total of 208,386 older people were included in the study, and the age-sex standardised prevalence of frailty and pre-frailty among older adults in China was 9.5% (95% CI 9.4–9.7) and 46.1% (45.9–46.3) respectively. The prevalence of frailty and pre-frailty was higher in female than in male older adults, higher in rural than in urban older adults, and higher in northern China than in southern China. The multinomial analysis revealed similar risk factors for frailty and pre-frailty, including increased age, being female, living in a rural area, low educational attainment, poor marital status, living alone, difficult financial status, poor access to medical reimbursement, and living in northern China.

**Conclusion:**

Frailty and pre-frailty are very common among older adults in China and differ significantly between southern and northern China, men and women, and rural and urban areas. Appropriate public health prevention strategies should be developed based on identified risk factors in frail and pre-frail populations. The management of frailty and pre-frailty should be optimised according to regional and gender differences in prevalence and associated factors, such as strengthening the integrated management of chronic diseases, increasing reimbursement rates for medical costs, and focusing on vulnerable groups such as the disabled, economically disadvantaged, living alone and those with low literacy levels, in order to reduce the burden of frailty among older adults in China.

## Introduction

Frailty is one of the common geriatric syndromes and a global emerging disease burden. It is characterized by a persistent, non-specific pathological state in which the functional reserve capacity of the older adults decreases, resulting in significantly increased vulnerability and functional decline against stressful conditions. Frailty not only increases the risk of falls, disability, hospitalization, and death in older adults, but also increases the need for long-term care and medical costs, thus seriously impacting their quality of life and healthy life expectancy (1–6). China has largest older population in the world, according to the seventh census, China has 264 million people aged 60 or above, accounting for 18.7% of the total population, China has entered an aging society (7). Frailty is an age-related geriatric syndrome. Studies have reported that the prevalence of frailty and pre-frailty in the older Chinese population was 5.9%–67.6% and 26.8%–62.8%, respectively (8). The frailty of the older adults has brought huge challenges to the developing Chinese older adults care system. However, the prevalence estimates from these studies are highly inconsistent, and these inconsistencies require further researches to generate more accurate estimates. These inconsistencies may due to the differences in the physical and geographical environment, uneven economic and health development, different habits of the population and varied prevalence of chronic diseases in different regions of China. Therefore, further research is needed to accurately assess the prevalence of frailty and pre-frailty in older adults and their risk factors in different regions.

We used data from the Fourth Sample survey of the Aged Population in Urban and Rural China (SSAPUR) conducted in 2015, which was a cross-sectional study in a nationally representative sample (*n* = 224,142) of older adults (aged ≥60 years) from 31 provinces, autonomous regions and municipalities in mainland China (9), to investigate regional differences in the prevalence of frailty and pre-frailty among the older adults in China and to identify associated factors. Such comparisons are more meaningful for identifying underlying causes of regional differences in frailty prevalence and designing corresponding public health interventions.

## Methods

### Study design and participants

The data for thi study were obtained from the 4th SSAPUR database, which is a national survey of older adults conducted by the National Committee on Ageing in China between August 1, 2015 and August 31, 2015, and included Chinese citizens aged ≥60 years residing in mainland China. The survey adopted a sampling design of “stratified, multi-stage, probability proportionate to size sampling, final stage equal probability.” The sampling ratio determined in the fourth SSAPUR survey was 1/1000 of the older population in 2015. The sampling for this study was conducted in four stages. In the first stage, the number of samples was allocated based on the proportion of the older population in each province/municipalities/autonomous regions in the mainland China. Four hundred sixty-six districts (counties) were selected from 2,853 districts (counties) in 31 provinces/municipalities/autonomous regions as primary sampling units. In the second stage, 4 townships (sub-districts) were selected in each district (county) according to the PPS sampling method based on the total number of older adults in each district (county). In the third stage, 4 village (residential) committees were selected in each townships (sub-districts) using the PPS sampling method based on the total number of older adults in each townships (sub-districts). In the fourth stage, 30 older adults were selected from each selected townships (sub-districts) using equidistant sampling based on the list of the older adults reported before the survey. The design sample size of the survey was 223,680 and the sampling ratio was about one-thousandth. Data for the fourth SSAPUR survey was collected by means of household interviews and questionnaires, with a simplified form used for 90% of the participants and a detailed form for 10%. Participants who declined to accept a visit, died, relocated, could not be contacted (after at least 3 attempts), or lived in a long-term older adults’ care institutions would be excluded, and a new participant was then selected in order from the candidate list. The survey covered nine aspects, including demographic information, family situation, health status, health care and nursing services, economic status, social activity, living environment, and spiritual and cultural life (including psychological status). The structure and sampling method of SSAPUR have been previously described in other studies (9–11). The design sample size of the survey was 223,680, and its actual collected samples were 224,142. SSAPUR is by far the largest database of older adults in China. The study protocol was approved by National Bureau of Statistics [No. (2014) 87] and the ethics committee of Beijing Hospital (2021BJYYEC-294-01). All participants provided written informed consent before participating in the survey.

**Figure 1:**
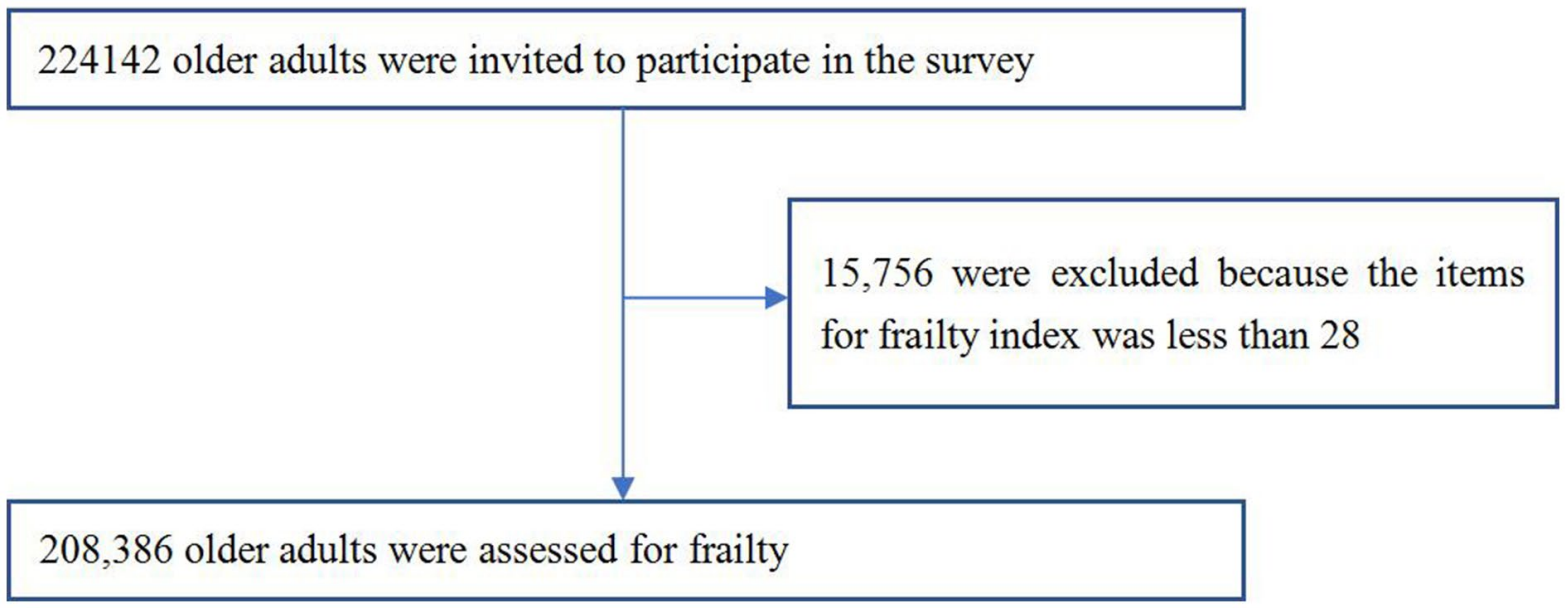
Flowchart of participants through the study.

Currently, there is no standardized assessment of frailty, and the two main methods widely used to assess frailty are the frailty phenotype assessment proposed by Fried et al. and the frailty index (FI) created by Professor Kenneth Rockwood’s team in Canada (1, 12). For the first time, the FI assessment method has succeeded in providing a quantitative description of frailty in older people and a broader assessment of frailty. A study comparing different frailty assessment tools applied to the same inpatient cohort showed that FI may be a superior assessment tool (13). Studies have confirmed that FI-based assessment of frailty is a good predictor of poor prognosis, including death in older adults (14, 15). Additionaly, previous studies have also shown good reliability and validity of the FI in Chinese populations (15–17). For this study, the construction of the FI included 33 items, but respondents needed at least 28/33 items to be included in this study, and a total of 208,386 people were eventually included in the analysis sample (see Figure 1).

### Demographics

Demographic characteristics include age (60–64, 65–69, 70–74, 75–79, 80–84, and ≥85 years), sex, education (primary school or lower, middle school, high school or higher), marital status (being married, widowed/divorced/unmarried), ethnicity (Han, Ethnic Minorities), current residence location (urban, rural), living status (living alone, not living alone), economic status (rich, adequate, poor), convenience of medical cost reimbursement (being convenient, less convenient, inconvenient), activities of daily living (ADL) disability (Inability to do one or more of the following is considered a disability: bathing, dressing, toileting, getting in and out of bed, eating and moving around the room), multimorbidity (combination of ≥2 chronic diseases), and northern China (including 3 administrative regions of northwest, north and northeast) and southern China (including 4 administrative regions of central, southwest, south and southeast).

### Identification and assignment of health deficit variables for FI

We constructed FI based on Searle’s standard procedure (18). The FI items (*n* = 33, each subject needs at least 28/33 items) were selected from the baseline questionnaires. The FI included eight items (bathing, dressing, toileting, getting in and out of bed, eating, walking around the room, urinary incontinence, and fecal incontinence) of basic ADL; ten items focusing on chronic diseases including glaucoma/cataract, cardiovascular disease, hypertension, diabetes, gastric disease, bone and joint disease, chronic lung disease, asthma, malignancy, and reproductive system disease; two items focusing on feelings of loneliness and happiness; three items focusing on geriatric syndrome, including visual impairment, hearing impairment, and history of falls; five items focusing on assistive devices (hearing aids, dentures, crutches, wheel-chairs, and adult diapers/nursing pads); three items focusing on mobility (needing care from others in daily life, self-rated health status, and exercise); two items focusing on social activity (regular leisure activities and regular public service activities). The FI was calculated by summing the number of deficits recorded for a patient and dividing this by the total number of possible deficits [the denominator of the FI was adjusted based on the number of questions answered (i.e., 28–33)]. An FI ≥0.25 indicates frailty, an FI <0.12 indicates robust older adults, and an FI between 0.12 and 0.25 indicates pre-frailty. The exact construction method has been described in our previous study (10).

### Statistical analysis

We weighted all calculations to represent the general adult population aged 60 years or older in China, according to the 2020 population census. We calculated weights using data from the census and study sample. To determine the sample size, we assumed a mean prevalence of 10% (SD 1.0) for frailty in people aged 60 years or older based on available data from previous studies (8). We used PASS software (NCSS, Kaysville, UT, United States) to calculate sample sizes and applied a design effect of 3 to account for the multistage cluster sampling design. The resulting sample size was 10,671. Our sample of over 200,000 participants in this study was therefore adequate.

We calculated the prevalence of frailty and pre-frailty separately for the overall population and for subgroups stratified by age, sex, education, urban and rural, ethnicity, marital status, living alone, medical insurance, ease of medical reimbursement, economic status, multimorbidity, disability, province/municipality/autonomous region, administrative region, and southern and northern China. Age-sex standardised prevalence was calculated using China’s population distribution in 2020 (7). We also calculated the absolute number of people with frailty and pre-frailty based on the 2020 Chinese population. Our analysis used all participants for whom the variable of interest was available, and we did not impute missing data.

We used one-way analysis of variance or Student’s t test for continuous variables and *χ*^2^ test for categorical variables to assess significant differences. The prevalence trend by covariables was tested using the Cochran–Armitage test. Multinomial logistic regression models were applied to ascertain the factors associated with frailty and pre-frailty, including age group (60–64, 65–69, 70–74, 75–79, 80–84, and ≥85 years), sex, ethnicity (Han and ethnic minorities), residence location (rural vs. urban), education level (elementary school and lower, middle school, High school and higher), marital status (married, widowed/divorced/unmarried), living alone (yes or no), economic status (rich, adequate, poor), medical insurance, convenience of medical expense reimbursement (convenient, less convenient, inconvenient), southern or northern China. All analyses were conducted with SPSS 24.0 (IBM Corp. Armonk, NY), and differences between groups were considered statistically significant for *p* < 0.05.

## Results

**Figure 2:**
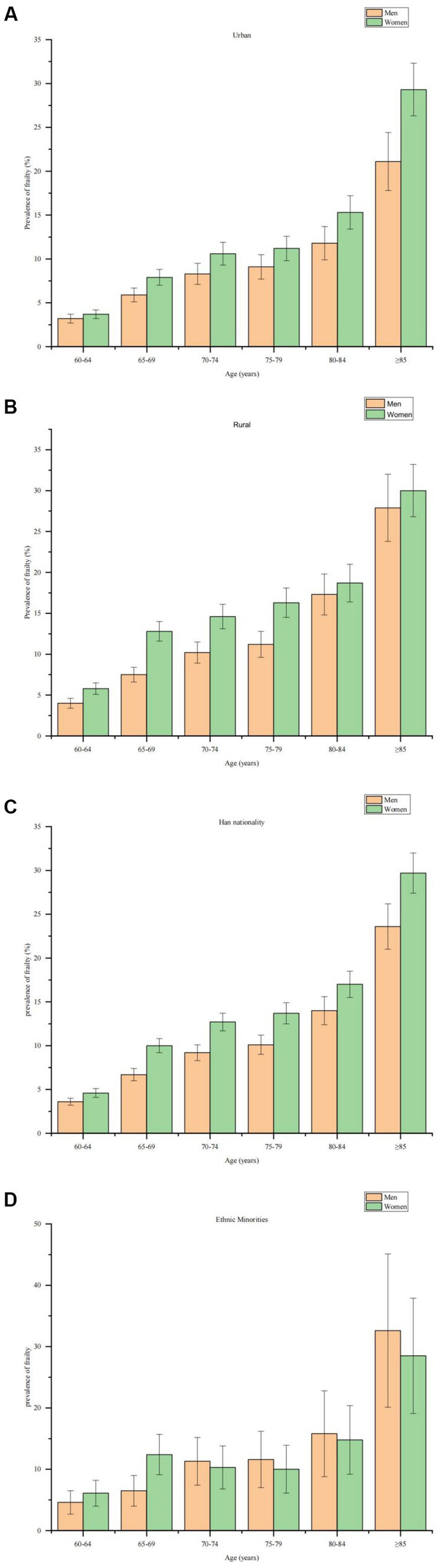
Age-sex standardized prevalence of frailty in older adults. **(A)** Age-sex standardized prevalence of frailty in older men and women living in urban areas. **(B)** Age-sex standardised prevalence of frailty in older men and women living in rural areas. **(C)** Age-sex standardised prevalence of frailty among older men and women of Han ethnicity. **(D)** Age-sex standardised prevalence of frailty among older men and women from ethnic minorities.

A total of 224,142 older adults aged 60 years or older were invited to participate in the fourth SSAPUR from 1 August 2015 to 31 August 2015, of which 15,756 participants were excluded because they had fewer than 28 items to construct the FI, leaving 208,386 older adults (99,586 men and 108,800 women) included in this study, of whom 19,609 were identified as frail (7,705 men and 11,904 women) and 95,453 were identified as pre-frail (42,955 men and 52,498 women). The basic characteristics of older adults by sex, northern and southern China, ethnicity, urban/rural subgroups are shown in Table 1. The demographics of the study population and the related factors for frailty by stage of frailty are shown in Table 2. The age-sex standardized prevalence of frailty and pre-frailty was 9.5% (95% CI 9.4–9.7) and 46.1% (45.9–46.3), with a lower age-sex standardized prevalence of frailty and pre-frailty in men than in women [7.9% (7.7–8.1) versus 11.0% (10.8–11.2); 43.8% (43.5–44.1) versus 48.1% (47.8–48.4), with a sex difference of *p* < 0.0001 for both]. This sex difference in frailty prevalence was found across all age groups in the general population and subgroups grouped by urban/rural, with the exception of ethnic minority subgropus, where among Han Chinese, the prevalence of frailty is more or less equal in men than in women (see Figure 2). The age-sex standardized prevalence of frailty and pre-frailty in adults aged 65 years or older was 11.3% (11.1–11.5) and 48.7% (48.4–48.9), respectively, with a higher prevalence of frailty and pre-frailty in woman than in men [13.1% (12.9-13.4) and 51.2% (50.9-51.6), versus 9.2% (9.0-9.5) and 45.8% (45.4-46.2), respectively]. The prevalence of frailty increased with age, ranging from 4.2% (4.0–4.3) among those aged 60–64 years to 27.4% (26.6–28.3) among those aged 85 years or older (Table 3). The prevalence of frailty and pre-frailty was higher in rural residents [10.8% (10.6–11.0) and 48.5% (48.2–48.8)] than in urban residents [8.3% (8.1–8.5) and 43.7% (43.4–44), respectively; both *p* < 0.0001 for urbanisation difference; Table 3]. We estimated that 25.15 (95% CI 24.82–25.49) million people aged 60 years or older in China have frailty, 121.64 (121.08–122.21) million with pre-frailty in 2020.

**Table 1:** Demographics of the Chinese adults aged 60 years or older in 2015, and related factors for frailty, by sex, north–south, nationality and urban–rural subgroups.

	Total (*n* = 208,386)	Men (*n* = 99,586)	Women (*n* = 108,800)	*p* for difference	Northern (*n* = 50,257)	Southern (*n* = 158,129)	*p* for difference	Urban (*n* = 107,327)	Rural (*n* = 10,159)	*p* for difference	Han Chinese (*n* = 195,808)	Ethnic minorities (*n* = 12,369)	*p* for difference
Proportional of participants	100%	47.8%	52.2%		24.1%	75.9%		51.5%	48.5%		94.1%	5.9%	
Age (years)	69.7 ± 7.8	69.4 ± 7.6	70.1 ± 8.1	<0.0001	69.3 ± 7.6	69.9 ± 7.9	<0.0001	69.9 ± 8.0	69.5 ± 7.7	<0.0001	69.7 ± 7.9	69.8 ± 7.6	0.218
Sex							0.438			<0.0001			0.239
Women	108,800 (52.2%)				26,164 (52.1%)	82,636 (52.3%)		57,146 (53.2%)	51,654 (51.1%)		102,164 (52.2%)	6,521 (52.7%)	
Men	99,586 (47.8%)				24,093 (47.9%)	75,493 (47.7%)		50,181 (46.8%)	49,405 (48.9%)		93,644 (47.8%)	5,848 (47.3%)	
Age group				<0.0001			<0.0001			<0.0001			<0.0001
60–64	68,711 (33.0%)	33,610 (33.9%)^a^	34,883 (32.2%)^b^		17,335 (34.5%)^a^	51,376 (32.5%)^b^		34,719 (50.5%)^a^	33,992 (49.5%)^b^		64,812 (33.1%)^a^	3,834 (31.0%)^b^	
65–69	49,195 (23.6%)	24,194 (24.4%)^a^	24,851 (22.9%)^b^		12,096 (24.1%)^a^	37,099 (23.5%)^b^		24,869 (23.2%)^a^	24,326 (24.1%)^b^		46,077 (23.5%)^a^	3,065 (24.8%)^b^	
70–74	34,619 (16.6%)	16,689 (16.8%)^a^	17,811 (16.4%)^b^		8,386 (16.7%)^a^	26,233 (16.6%)^a^		17,709 (16.5%)^a^	16,910 (16.7%)^a^		32,384 (16.5%)^a^	2,206 (17.8%)^b^	
75–79	27,260 (13.1%)	12,739 (12.8%)^a^	14,432 (13.3%)^b^		6,479 (12.9%)^a^	20,781 (13.1%)^a^		14,284 (13.3%)^a^	12,976 (12.8%)^b^		25,570 (13.1%)^a^	1,664 (13.5%)^b^	
80–84	17,862 (8.6%)	7,824 (7.9%)^a^	9,985 (9.2%)^b^		3,925 (7.8%)^a^	13,937 (8.8%)^b^		9,928 (9.3%)^a^	7,934 (7.9%)^b^		16,808 (8.6%)^a^	1,030 (8.3%)^a^	
≥85	10,739 (5.2%)	4,202 (4.2%)^a^	6,495 (6.0%)^b^		2,036 (4.1%)^a^	8,703 (5.5%)^b^		5,818 (5.4%)^a^	4,921 (4.9%)^b^		10,157 (5.2%)^a^	570 (4.6%)^b^	
Urban or rural area				<0.0001			0.299						<0.0001
Urban	107,327 (51.5%)	50,181 (50.4%)	57,146 (52.5%)		25,783 (51.3%)	81,544 (51.6%)					102,689 (52.4%)	4,547 (36.8%)	
Rural	101,059 (48.5%)	49,405 (49.6%)	51,654 (47.5%)		24,474 (48.7%)	76,585 (48.4%)					93,119 (47.6%)	7,822 (63.2%)	
Education				<0.0001			<0.0001			<0.0001			<0.0001
Primary school or less	148,670 (71.6%)	60,591 (61.0%)^a^	88,079 (81.2%)^b^		31,913 (63.7%)^a^	116,757 (74.1%)^b^		63,799 (59.6%)^a^	84,871 (84.3%)^b^		138,850 (71.1%)^a^	9,670 (78.6%)^b^	
Middle school	38,517 (18.5%)	25,055 (25.2%)^a^	13,462 (12.4%)^b^		11,579 (23.1%)^a^	26,938 (17.1%)^b^		25,155 (23.5%)^a^	13,362 (13.3%)^b^		36,742 (18.8%)^a^	1,750 (14.2%)^b^	
High school or higher	20,528 (9.9%)	13,612 (13.7%)^a^	6,916 (6.4%)^b^		6,596 (13.2%)^a^	13,932 (8.8%)^b^		18,081 (16.9%)^a^	2,447 (2.4%)^b^		19,629 (10.1%)^a^	890 (7.2%)^b^	
Marital status				<0.0001			0.0079			<0.0001			<0.0001
Married	147,948 (72.1%)	79,770 (81.3%)	68,178 (63.6%)		35,951 (72.7%)	111,997 (71.9%)		77,778 (73.4%)	70,170 (70.7%)		139,710 (72.4%)	8,105 (66.7%)	
Others	57,303 (27.9%)	18,318 (18.7%)	38,985 (36.4%)		13,520 (27.3%)	43,783 (28.1%)		28,244 (26.6%)	29,059 (29.3%)		53,207 (27.6%)	4,045 (33.3%)	
Ethnicity				0.239			<0.0001			<0.0001			
Han	195,808 (94.1%)	93,644 (94.1%)	102,164 (94.0%)		46,743 (93.1%)	149,065 (94.4%)		102,689 (95.8%)	93,119 (92.3%)				
Non-Han	12,369 (5.9%)	5,848 (5.9%)	6,521 (6.0%)		3,444 (6.9%)	8,925 (5.6%)		4,547 (4.2%)	7,822 (7.7%)				
Living status				<0.0001			<0.0001			<0.0001			<0.0001
Living alone	28,036 (13.5%)	11,256 (11.3%)	16,780 (15.4%)		6,290 (12.5%)	21,746 (13.8%)		13,203 (12.3%)	14,833 (14.7%)		26,798 (13.7%)	1,202 (9.7%)	
Not living alone	179,970 (86.5%)	88,110 (88.7%)	91,860 (84.6%)		43,851 (87.5%)	136,119 (86.2%)		93,979 (87.7%)	85,991 (85.3%)		168,650 (86.3%)	11,150 (90.3%)	
Economic status				0.178			<0.0001			<0.0001			<0.0001
Rich	33,413 (16.1%)	15,989 (16.2%)	17,424 (16.1%)		7,325 (14.7%)^a^	26,088 (16.6%)^b^		20,874 (19.6%)^a^	12,539 (12.5%)^b^		31,849 (16.4%)^a^	1,537 (12.5%)^b^	
Adequate	121,097 (58.5%)	57,680 (58.3%)	63,417 (58.7%)		28,229 (56.7%)^a^	92,868 (59.1%)^b^		64,903 (60.9%)^a^	56,194 (56.0%)^b^		114,288 (58.8%)^a^	6,689 (54.5%)^b^	
Poor	52,405 (25.3%)	25,213 (25.5%)	27,192 (25.2%)		14,259 (28.6%)^a^	38,146 (24.3%)^b^		20,789 (19.5%)^a^	31,616 (31.5%)^b^		48,292 (24.8%)^a^	4,054 (33.0%)^b^	
Medicare				0.326			<0.0001			0.074			<0.0001
Yes	205,834 (99.1%)	98,332 (99.1%)	107,502 (99.1%)		49,540 (98.9%)	1,566,294 (99.2%)		106,094 (99.1%)	99,740 (99.1%)		193,437 (99.1%)	12,188 (98.8%)	
No	1,848 (0.9%)	904 (0.9%)	944 (0.9%)		554 (1.1%)	1,294 (0.8%)		914 (0.9%)	934 (0.9%)		1,694 (0.9%)	154 (1.2%)	
Convenience of medical cost reimbursement				0.0017			<0.0001			<0.0001			0.0059
Convenient	147,063 (74.7%)	70,022 (74.5%)^a^	77,041 (74.9%)^b^		33,927 (71.7%)^a^	113,136 (75.7%)^b^		77,911 (76.6%)^a^	69,152 (72.6%)^b^		138,333 (74.8%)^a^	8,587 (73.5%)^b^	
Less convenient	38,047 (19.3%)	18,235 (19.4%)^a^	19,812 (19.3%)^a^		9,530 (20.1%)^a^	28,517 (19.1%)^b^		18,252 (18.0%)^a^	19,795 (20.8%)^b^		35,652 (19.3%)^a^	2,359 (20.2%)^b^	
Inconvenient	11,737 (6.0%%)	5,786 (6.2%)^a^	5,951 (5.8%)^b^		3,869 (8.2%)^a^	7,868 (5.3%)^b^		5,491 (5.4%)^a^	6,246 (6.6%)^b^		10,980 (5.9%)^a^	741 (6.3%)^a^	
Comorbidities				<0.0001			<0.0001			0.388			0.875
<2	104,820 (50.3%)	54,220 (54.4%)	50,600 (46.5%)		22,860 (45.5%)	81,960 (51.8%)		53,888 (50.2%)	50,932 (50.4%)		98,481 (50.3%)	6,230 (50.4%)	
≥2	103,566 (49.7%)	45,366 (45.6%)	58,200 (53.5%)		27,397 (54.5%)	76,169 (48.2%)		53,439 (49.8%)	50,127 (49.6%)		97,327 (49.6%)	6,139 (49.6%)	
ADL disability				<0.0001			<0.0001			<0.0001			<0.0001
Yes	8,737 (4.2%)	3,490 (3.5%)	5,247 (4.8%)		3,320 (6.6%)	5,417 (3.4%)		4,307 (4.0%)	4,430 (4.4%)		8,016 (4.1%)	710 (5.7%)	
No	199,649 (95.8%)	96,096 (96.5%)	103,553 (95.2%)		46,937 (93.4%)	152,712 (96.6%)		103,020 (96.0%)	96,629 (95.6%)		187,792 (95.9%)	11,659 (94.3%)	
Southern or nortnern				0·438						0.299			<0.0001
Northern	50,257 (24.1%)	24,093 (24·2%)	26,164 (24·0%)					25,783 (24.0%)	24,474 (24.2%)		46,743 (23.9%)	3,444 (27.8%)	
Southern	158,129 (75.9%)	75,493 (75·8%)	82,636 (76·0%)					81,544 (76.0%)	76,585 (75.8%)		149,065 (76.1%)	8,925 (72.2%)	

ADL, activities of daily living. The superscript letters a, b indicate the difference in the demographics among different subgroups (adjusted *p*-values).

**Table 2:** Demographics of the Chinese adults aged 60 years or older in 2015, and related factors for frailty, by frailty stage.

	Men				Women			
	Robust (48,923)	Pre-frailty (42,958)	Frailty (7,705)	*p* for difference	Robust (44,396)	Pre-frailty (52,500)	Frailty (11,904)	*p* for difference
Proportion of participants	49.1%	43.1%	7.7%	<0.0001	40.8%	48.3%	10.9%	<0.0001
Age (years)	67.9 ± 6.9	70.3 ± 7.7	73.6 ± 8.5	<0.0001	68.1 ± 7.3	70.8 ± 8.1	74.3 ± 8.9	<0.0001
Age group				<0.0001				<0.0001
60–64	19,852 (40.7%)	12,354 (28.9%)	1,404 (18.3%)		18,194 (41.1%)	14,668 (28.0%)	2021 (17.0%)	
65–69	12,585 (25.8%)	10,208 (23.8%)	1,401 (18.2%)		10,867 (24.6%)	11,879 (22.7%)	2,105 (17.7%)	
70–74	7,481 (15.3%)	7,832 (18.3%)	1,376 (17.9%)		6,543 (14.8%)	9,279 (17.7%)	1,989 (16.7%)	
75–79	4,973 (10.2%)	6,338 (14.8%)	1,428 (18.6%)		4,558 (10.3%)	7,716 (14.7%)	2,158 (18.2%)	
80–84	2,695 (5.5%)	3,948 (9.2%)	1,181 (15.4%)		2,610 (5.9%)	5,456 (10.4%)	1,919 (16.2%)	
≥85	1,173 (2.4%)	2,139 (5.0%)	890 (11.6%)		1,476 (3.3%)	3,333 (6.4%)	1,686 (14.2%)	
Urban or rural residents				<0.0001				<0.0001
Urban residents	26,305 (53.8%)	20,356 (47.4%)	3,520 (45.7%)		25,196 (56.8%)	26,479 (50.4%)	5,471 (46.0%)	
Rural residents	22,618 (46.2%)	22,602 (52.6%)	4,185 (54.3%)		19,200 (43.2%)	26,021 (49.6%)	6,433 (54.0%)	
Education level				<0.0001				<0.0001
Primary school or less	27,628 (56.7%)	27,540 (64.3%)	5,423 (70.6%)		34,119 (77.1%)	43,427 (83.0%)	10,533 (88.7%)	
Middle school	13,593 (27.9%)	9,947 (23.2%)	1,515 (19.7%)		6,694 (15.1%)	5,887 (11.2%)	881 (7.4%)	
High school or higher	7,538 (15.5%)	5,332 (12.5%)	742 (9.7%)		3,435 (7.8%)	3,017 (5.8%)	464 (3.9%)	
Ethnicity				<0.0001				<0.0001
Han	46,156 (94.4%)	40,285 (93.9%)	7,203 (93.6%)		41,872 (94.4%)	49,120 (93.7%)	11,172 (93.9%)	
Ethnic minorities	2,725 (5.6%)	2,630 (6.1%)	493 (6.4%)		2,470 (5.6%)	3,329 (6.3%)	722 (6.1%)	
Marital status				<0.0001				<0.0001
Married	41,885 (87.0%)	32,567 (76.9%)	5,318 (70.0%)		32,059 (73.4%)	30,674 (59.2%)	5,445 (46.4%)	
Others	6,242 (13.0%)	9,802 (23.1%)	2,274 (30.0%)		11,602 (26.6%)	21,100 (40.8%)	6,283 (53.6%)	
Living status				<0.0001				<0.0001
Living alone	3,127 (6.4%)	6,758 (15.8%)	1,371 (17.8%)		3,410 (7.7%)	10,489 (20.0%)	2,881 (24.2%)	
Not living alone	45,689 (93.6%)	36,108 (84.2%)	6,313 (82.2%)		40,909 (92.3%)	41,949 (80.0%)	9,002 (75.8%)	
Medical insurance				0.594				0.257
No	433 (0.9%)	394 (0.9%)	77 (1.0%)		362 (0.8%)	469 (0.9%)	113 (1.0%)	
Yes	48,311 (99.1%)	42,423 (99.1%)	7,598 (99.0%)		43,877 (99.2%)	51,873 (99.1%)	11,752 (99.0%)	
Convenience of medical expense reimbursement				<0.001				<0.001
Convenience	35,207 (76.3%)	29,618 (72.9%)	5,197 (71.3%)		32,142 (76.7%)	36,782 (74.0%)	8,117 (72.2%)	
Still acceptable	8,590 (18.6%)	8,206 (20.2%)	1,439 (19.8%)		7,835 (18.7%)	9,758 (19.6%)	2,219 (19.7%)	
Inconvenience	2,355 (5.1%)	2,781 (6.8%)	650 (8.9%)		1,915 (4.6%)	3,135 (6.3%)	901 (8.0%)	
Economic status				<0.0001				<0.0001
Rich	9,648 (19.9%)	5,547 (13.0%)	794 (10.4%)		9,094 (20.6%)	7,096 (13.6%)	1,234 (10.4%)	
Adequate	29,880 (61.5%)	23,970 (56.2%)	3,830 (50.1%)		27,497 (62.4%)	29,886 (57.3%)	6,034 (51.0%)	
Poor	9,039 (18.6%)	13,154 (30.8%)	3,020 (39.5%)		7,483 (17.0%)	15,144 (29.1%)	4,565 (38.6%)	
Living in northern or southern China				<0.0001				<0.0001
Northern	10,294 (21.0%)	11,181 (26.0%)	2,618 (34.0%)		8,963 (20.2%)	13,299 (25.3%)	3,902 (32.8%)	
Southern	38,629 (79.0%)	31,777 (74.0%)	5,087 (66.0%)		35,433 (79.8%)	39,201 (74.7%)	8,002 (67.2%)	
Comorbidity				<0.0001				<0.0001
≥2	7,623 (15.6%)	30,824 (71.8%)	6,919 (89.8%)		8,062 (18.2%)	39,330 (74.9%)	10,808 (90.8%)	
<2	41,300 (84.4%)	12,134 (28.2%)	786 (10.2%)		36,334 (81.8%)	13,170 (25.1%)	1,096 (9.2%)	
ADL disability				<0.0001				<0.0001
Yes	70 (0.1%)	923 (2.1%)	2,497 (32.4%)		70 (0.2%)	1,420 (2.7%)	3,757 (31.6%)	
No	48,853 (99.9%)	42,035 (97.9%)	5,208 (67.6%)		44,326 (99.8%)	51,080 (97.3%)	8,147 (68.4%)	

ADL, activities of daily living.

**Table 3:** Age-sex standardized prevalence of frailty and pre-frailty among people aged 60 years or older in China, 2015.

	Prevalence (%) of pre-frailty (95% CI)			Prevalence (%) of frailty (95% CI)		
	Men	Women	Total	Men	Women	Total
	43.8% (43.5–44.1)	48.1% (47.8–48.4)	46.1% (45.9–46.3)	7.9% (7.7–8.1)	11.0% (10.8–11.2)	9.5% (9.4–9.7)
*Age (years)*						
60–64	32.1% (31.6–32.6)	34.1% (33.6–34.6)	33.1% (32.7–33.5)	3.7% (3.5–3.9)	4.7% (4.5–4.9)	4.2% (4.0–4.3)
65–69	49.1% (48.5–49.7)	57.8% (57.2–58.4)	53.5% (52.9–54.1)	6.8% (6.4–7.1)	10.3% (9.9–10.7)	8.5% (8.3–8.8)
70–74	53.3% (52.6–54.1)	58.7% (58.0–59.5)	56.1% (55.4–56.9)	9.3% (8.9–9.8)	12.5% (12.0–13.0)	11.0% (10.6–11.3)
75–79	45.4% (44.5–46.3)	48.1% (47.3–48.9)	46.8% (46.1–47.6)	10.2% (9.7–10.7)	13.4% (12.9–14.0)	11.9% (11.5–12.3)
80–84	47.0% (45.9–48.1)	48.0% (47.0–49.0)	47.5% (46.6–48.5)	14.1% (13.3–14.8)	16.8% (16.1–17.6)	15.6% (15.1–16.1)
≥85	58.3% (56.8–59.7)	58.1% (56.9–59.3)	58.2% (56.9–59.4)	24.3% (23.0–25.6)	29.4% (28.3–30.5)	27.4% (26.6–28.3)
Urban or rural residents	<0.0001	<0.0001	<0.0001	<0.0001	<0.0001	<0.0001
Rural	45.5% (45.1–46)	51.3% (50.9–51.8)	48.5% (48.2–48.8)	8.6% (8.3–8.8)	12.9% (12.6–13.2)	10.8% (10.6–11.0)
Urban	41.9% (41.5–42.4)	45.3% (44.9–45.7)	43.7% (43.4–44.0)	7.1% (6.9–7.4)	9.3% (9.1–9.6)	8.3% (8.1–8.5)
*p* for difference	<0.0001	<0.0001	<0.0001	<0.0001	<0.0001	<0.0001
*Education level*						
Primary school or less	45.5% (45.1–45.9)	48.9% (48.5–49.2)	47.5% (47.3–47.8)	8.6% (8.4–8.8)	11.6% (11.4–11.8)	10.4% (10.2–10.5)
Middle school	41.9% (41.3–42.5)	46.3% (45.4–47.1)	43.5% (43.0–44.0)	7.3% (7.0–7.7)	8.2% (7.7–8.7)	7.6% (7.4–7.9)
High school or higher	39.8% (38.9–40.6)	44.8% (43.6–45.9)	41.4% (40.8–42.1)	5.7% (5.3–6)	8.1% (7.4–8.7)	6.5% (6.1–6.8)
*p* for trend	<0.0001	<0.0001	<0.0001	<0.0001	<0.0001	<0.0001
*Ethnicity*						
Ethnic minorities	46.1% (44.9–47.4)	50.1% (48.9–51.3)	48.2% (47.4–49.1)	8.8% (8.1–9.6)	11.0% (10.2–11.8)	10.0% (9.5–10.5)
Han	43.7% (43.4–44.0)	48.0% (47.7–48.3)	46.0% (45.7–46.2)	7.8% (7.6–8)	11.0% (10.8–11.2)	9.5% (9.3–9.6)
*p* for difference	0.0003	0.0010	<0.0001	0.0058	0.9832	0.0663
*Marital status*						
Widowed, divorced, and unmarried	53.5% (52.8–54.2)	52.8% (52.3–53.2)	53.0% (52.6–53.4)	10.2% (9.7–10.6)	13.0% (12.7–13.4)	12.1% (11.8–12.4)
Married	42.1% (41.7–42.4)	46.1% (45.7–46.5)	43.9% (43.7–44.2)	7.3% (7.1–7.5)	9.5% (9.3–9.7)	8.3% (8.2–8.5)
*p* for difference	<0.0001	<0.0001	<0.0001	<0.0001	<0.0001	<0.0001
*Living status*						
Living alone	71.7% (70.9–72.5)	53.6% (52.8–54.3)	60.8% (60.3–61.4)	13.3% (12.7–13.9)	13.3% (12.8–13.8)	13.3% (12.9–13.7)
Not living alone	40.8% (40.4–41.1)	46.9% (46.5–47.2)	43.9% (43.7–44.1)	7.3% (7.1–7.5)	10.5% (10.3–10.7)	9.0% (8.8–9.1)
*p* for difference	<0.0001	<0.0001	<0.0001	<0.0001	<0.0001	<0.0001
*Economic status*						
Poor	52.5% (51.9–53.1)	55.8% (55.2–56.4)	54.2% (53.8–54.6)	12.3% (11.9–12.7)	17.0% (16.6–17.5)	14.8% (14.5–15.1)
Adequate	42.4% (42–42.8)	46.9% (46.5–47.3)	44.8% (44.5–45)	6.8% (6.6–7)	9.5% (9.3–9.7)	8.2% (8.1–8.4)
Rich	35.1% (34.4–35.8)	40.6% (39.9–41.4)	38.0% (37.5–38.5)	4.9% (4.6–5.3)	7.1% (6.7–7.5)	6.1% (5.8–6.3)
*p* for trend	<0.0001	<0.0001	<0.0001	<0.0001	<0.0001	<0.0001
*Medical insurance*						
No	43.2% (40–46.5)	50.5% (47.3–53.6)	46.9% (44.7–49.2)	8.7% (6.9–10.5)	12.4% (10.3–14.5)	10.6% (9.2–12)
Yes	43.8% (43.5–44.2)	48.1% (47.8–48.4)	46.1% (45.8–46.3)	7.9% (7.7–8.1)	11.0% (10.8–11.2)	9.5% (9.4–9.6)
*p* for difference	0.7174	0.1417	0.4922	0.3749	0.1713	0.1085
*Convenience of medical expense reimbursement*						
Inconvenience	47.4% (46.2–48.7)	54.1% (52.9–55.4)	50.8% (49.9–51.7)	11.1% (10.3–12)	15.8% (14.8–16.7)	13.5% (12.9–14.1)
Still acceptable	45.6% (44.9–46.4)	49.2% (48.5–49.9)	47.5% (47–48)	8.1% (7.7–8.5)	11.3% (10.8–11.7)	9.8% (9.5–10.1)
Convenience	43.1% (42.7–43.5)	47.4% (47.1–47.8)	45.4% (45.1–45.6)	7.6% (7.4–7.8)	10.5% (10.3–10.7)	9.1% (9–9.3)
*p* for trend	<0.0001	<0.0001	<0.0001	<0.0001	<0.0001	<0.0001
*ADL disability*						
Yes	27.5% (26.1–29)	26.7% (25.5–27.9)	27.0% (26.1–28)	70.3% (68.8–71.8)	71.1% (69.8–72.3)	70.7% (69.8–71.7)
No	44.6% (44.3–44.9)	49.4% (49.1–49.7)	47.1% (46.9–47.3)	5.6% (5.5–5.8)	8.0% (7.9–8.2)	6.9% (6.8–7)
*p* for difference	<0.0001	<0.0001	<0.0001	<0.0001	<0.0001	<0.0001
*Comorbidity*						
≥2	75.0% (74.6–75.4)	62.2% (61.8–62.6)	67.8% (67.5–68.1)	16.4% (16–16.7)	17.0% (16.7–17.3)	16.7% (16.5–16.9)
<2	21.5% (21.2–21.9)	28.9% (28.5–29.3)	25.1% (24.8–25.3)	1.5% (1.4–1.6)	2.5% (2.3–2.6)	1.9% (1.9–2)
*p* for difference	<0.0001	<0.0001	<0.0001	<0.0001	<0.0001	<0.0001

ADL, activities of daily living; CI, confidence interval.

The prevalence of pre-frailty was higher in ethnic minorities [48.2% (47.4–49.1)] than in Han Chinese [46.0% (45.7–46.2)], with no difference in the prevalence of frailty between the two groups. The prevalence of frailty and pre-frailty was higher among older people with poor marital status (widowed/divorced/unmarried) than among married older people; the prevalence of frailty and pre-frailty was lower among older adults with higher levels of education; the prevalence of frailty and pre-frailty was higher among older adults living alone than among older adults not living alone; the prevalence of frailty and pre-frailty was lower among older adults with better financial status; the prevalence of frailty and pre-frailty was higher among older adults with greater difficulty in reimbursing medical expenses; the prevalence of frailty was higher among older adults with multiple morbidities than those without; and the prevalence of frailty was higher among older adults with disabilities than those without disabilities, while no difference was observed in the prevalence of frailty and pre-frailty among older adults with and without health insurance (see Table 3).

The age-sex standardized prevalence of frailty and pre-frailty among older adults varied significantly among provinces/autonomous regions/municipalities (both *p* < 0.0001). The prevalence of frailty ranged approximately five folds from 4.4% (3.9–5) in Fujian Province to 21.4% (20.0–22.8) in Inner Mongolia Autonomous Region, and age-sex standardized pre-frailty prevalence ranged from 33.4% (32.4–34.5) in Jiangsu Province to 66.1% (61.4–70.8) in Xizang Autonomous Region (see Table 4). Significant differences in the age-sex standardized prevalence of frailty and pre-frailty were also observed among older adults in different administrative regions of China, with the highest age-sex standardized prevalence of frailty observed in northwest China 14.5% (13.9–15.1), followed by north 13.8% (13.4–14.2), northeast 11.9% (11.3–12.4), southwest 10.8% (10.5–11.1), and central China 10.4% (10–10.7), the lowest age-sex standardized prevalence of frailty were observed in southeast 6.9% (6.7–7.1) and south China 5.7% (5.4–6.1) (see Table 4). The age-sex standardized prevalence of frailty and pre-frailty among older adults was significantly higher in the northern China compared with the southern China [13.5% (13.2–13.8) vs. 8.3% (8.1–8.4), and 49.1% (48.6–49.5) vs. 45.1% (44.8–45.3), respectively; both *p* < 0.0001] (see Table 4).

**Table 4:** Age-sex standardized prevalence of frailty and pre-frailty among people aged 60 years or older among different province/municipality/autonomous region, administrative region, and southern and northern China.

	Prevalence (%) of pre-frailty (95% CI)			Prevalence (%) of frailty (95% CI)		
	Men	Women	Total	Men	Women	Total
*Northern or southern China*						
Northern	47.1% (46.4–47.7)	50.9% (50.3–51.5)	49.1% (48.6–49.5)	11.2% (10.8–11.6)	15.6% (15.2–16.1)	13.5% (13.2–13.8)
Southern	42.7% (42.4–43.1)	47.2% (46.9–47.5)	45.1% (44.8–45.3)	6.8% (6.7–7)	9.6% (9.4–9.8)	8.3% (8.1–8.4)
*p* for difference	<0.0001	<0.0001	<0.0001	<0.0001	<0.0001	<0.0001
*Administrative district*						
North China	47.4% (46.5–48.3)	49.8% (48.9–50.7)	48.7% (48–49.3)	11.8% (11.2–12.4)	15.6% (15–16.3)	13.8% (13.4–14.2)
Northeast China	41.7% (40.5–42.9)	48.0% (46.8–49.2)	44.9% (44–45.8)	9.7% (8.9–10.4)	14.0% (13.1–14.8)	11.9% (11.3–12.4)
Southeast China	39.7% (39.2–40.2)	45.2% (44.7–45.7)	42.6% (42.2–42.9)	5.7% (5.5–6)	8.0% (7.7–8.3)	6.9% (6.7–7.1)
Central China	46.0% (45.2–46.8)	49.8% (49.1–50.6)	48.0% (47.4–48.6)	8.3% (7.8–8.7)	12.3% (11.7–12.8)	10.4% (10–10.7)
South China	39.6% (38.7–40.5)	43.1% (42.2–44)	41.4% (40.8–42.1)	5.0% (4.6–5.4)	6.5% (6–6.9)	5.7% (5.4–6.1)
Southwest China	47.7% (46.9–48.4)	51.1% (50.4–51.8)	49.5% (48.9–50)	8.9% (8.5–9.4)	12.5% (12–13)	10.8% (10.5–11.1)
Northwest China	51.5% (50.3–52.7)	55.5% (54.4–56.7)	53.6% (52.7–54.4)	11.8% (11–12.6)	17.1% (16.2–18)	14.5% (13.9–15.1)
*p* for trend	<0.0001	<0.0001	<0.0001	<0.0001	<0.0001	<0.0001
*Province, municipality, autonomous region*						
Anhui Province	44.3% (43–45.6)	54.8% (53.5–56.1)	49.5% (48.3–50.8)	7.5% (6.9–8.2)	12.2% (11.4–13.1)	9.9% (9.3–10.4)
Beijing	46.1% (43.6–48.6)	48.9% (46.6–51.2)	47.6% (45.4–49.8)	7.9% (6.5–9.2)	10.8% (9.4–12.2)	9.5% (8.5–10.4)
Fujian Province	35.6% (33.7–37.5)	41.5% (39.7–43.4)	38.7% (37.1–40.4)	3.9% (3.1–4.6)	4.9% (4.1–5.7)	4.4% (3.9–5)
Gansu Province	56.2% (53.9–58.6)	59.9% (57.6–62.2)	58.1% (55.8–60.4)	14.5% (12.8–16.2)	25.9% (23.8–28)	20.2% (18.9–21.6)
Guangdong Province	38.0% (36.8–39.2)	40.4% (39.3–41.6)	39.3% (38.3–40.3)	4.4% (3.9–4.9)	5.7% (5.2–6.2)	5.1% (4.7–5.5)
Guangxi Zhuang Autonomous Region	39.3% (37.8–40.9)	45.5% (44–47)	42.5% (41.1–43.9)	4.6% (3.9–5.2)	6.2% (5.4–6.9)	5.4% (4.9–5.9)
Guizhou Province	45.0% (43.2–46.9)	49.7% (47.9–51.5)	47.5% (45.8–49.2)	7.1% (6.1–8)	9.5% (8.4–10.6)	8.3% (7.6–9)
Hainan Province	56.3% (52.6–60)	59.0% (55.4–62.5)	57.7% (54–61.3)	12.4% (10–14.9)	14.6% (12.1–17.2)	13.6% (11.8–15.3)
Hebei Province	47.4% (46.1–48.8)	49.1% (47.8–50.4)	48.3% (47.1–49.6)	11.7% (10.8–12.6)	15.5% (14.6–16.4)	13.7% (13.1–14.4)
Henan Province	49.8% (48.6–51)	50.1% (49–51.2)	50.0% (48.9–51)	9.4% (8.7–10.1)	14.1% (13.3–14.9)	11.9% (11.4–12.5)
Heilongjiang Province	40.5% (38.7–42.3)	49.3% (47.4–51.1)	44.8% (43.2–46.5)	9.0% (7.9–10)	13.4% (12.2–14.7)	11.2% (10.3–12)
Hubei Province	42.8% (40.5–45.2)	48.7% (46.4–51)	45.8% (43.7–48)	6.5% (5.3–7.7)	10.0% (8.6–11.3)	8.3% (7.4–9.2)
Hunan Province	42.7% (41.4–44)	49.6% (48.3–50.8)	46.3% (45.1–47.4)	7.6% (6.9–8.3)	10.6% (9.8–11.3)	9.2% (8.6–9.7)
Jilin Province	46.8% (44.6–49)	50.2% (48.1–52.3)	48.6% (46.6–50.5)	12.7% (11.3–14.2)	16.9% (15.4–18.5)	14.9% (13.8–16)
Jiangsu Province	33.4% (32.4–34.5)	38.9% (37.8–39.9)	36.3% (35.4–37.2)	4.5% (4–5)	6.2% (5.7–6.7)	5.4% (5–5.7)
Jiangxi Province	40.1% (38.3–41.9)	44.9% (43.2–46.6)	42.6% (41.1–44.2)	6.4% (5.5–7.3)	7.6% (6.7–8.5)	7.1% (6.4–7.7)
Liaoning Province	36.0% (33.4–38.7)	41.8% (39.2–44.4)	39.0% (36.8–41.3)	6.4% (5.1–7.8)	10.1% (8.5–11.7)	8.3% (7.3–9.4)
Inner Mongolia Autonomous Region	49.4% (47–51.9)	51.2% (48.9–53.6)	50.4% (48.2–52.5)	18.9% (17–20.8)	23.7% (21.7–25.6)	21.4% (20–22.8)
Ningxia Hui Autonomous Region	48.6% (44.1–53)	58.5% (54.1–62.9)	53.5% (49.2–57.8)	11.1% (8.3–13.9)	19.2% (15.6–22.7)	15.1% (12.9–17.4)
Qinghai Province	53.7% (49.1–58.3)	58.5% (54.2–62.8)	56.3% (51.7–60.8)	10.7% (7.8–13.5)	9.0% (6.5–11.5)	9.8% (7.9–11.7)
Shandong Province	40.4% (39.4–41.5)	43.9% (42.9–44.9)	42.3% (41.4–43.2)	6.5% (6–7)	9.1% (8.5–9.7)	7.9% (7.5–8.3)
Shanxi Province	47.0% (45.1–49)	51.3% (49.4–53.2)	49.3% (47.5–51)	10.8% (9.6–12)	16.5% (15.1–17.9)	13.8% (12.8–14.7)
Shaanxi Province	52.5% (50.6–54.4)	51.9% (50.1–53.7)	52.2% (50.4–53.9)	9.8% (8.7–10.9)	13.6% (12.4–14.8)	11.8% (11–12.7)
Shanghai	40.8% (38.7–42.9)	43.5% (41.5–45.6)	42.2% (40.3–44.1)	5.0% (4.1–5.9)	7.3% (6.2–8.3)	6.2% (5.5–6.9)
Sichuan Province	46.8% (45.7–47.9)	50.6% (49.6–51.7)	48.8% (47.7–49.8)	9.3% (8.6–9.9)	13.8% (13–14.5)	11.6% (11.1–12.1)
Tianjin	45.6% (42.3–48.8)	46.9% (43.9–50)	46.3% (43.4–49.2)	8.7% (6.8–10.5)	9.5% (7.7–11.3)	9.1% (7.8–10.4)
Xizang Autonomous Region	66.1% (61.4–70.8)	51.0% (46.8–55.2)	57.3% (52.8–61.8)	12.2% (8.9–15.4)	16.8% (13.6–19.9)	14.8% (12.5–17.1)
Xinjiang Uygur Autonomous Region	42.9% (40.1–45.7)	55.9% (53.1–58.7)	49.4% (46.8–52)	12.3% (10.5–14.2)	17.3% (15.1–19.4)	14.8% (13.4–16.2)
Yunnan Province	53.2% (51.4–54.9)	53.1% (51.4–54.7)	53.1% (51.5–54.8)	11.0% (9.8–12.1)	12.4% (11.3–13.5)	11.7% (11–12.5)
Zhejiang Province	43.5% (42.1–44.9)	51.8% (50.4–53.2)	47.7% (46.4–49.1)	4.8% (4.1–5.4)	6.7% (6.0–7.4)	5.8% (5.3–6.2)
Chongqing	44.8% (43–46.5)	51.0% (49.3–52.8)	47.9% (46.3–49.6)	7.6% (6.6–8.5)	11.2% (10.1–12.3)	9.4% (8.7–10.1)
*p* for trend	<0.0001	<0.0001	<0.0001	<0.0001	<0.0001	<0.0001

ADL, activities of daily living; CI, confidence interval.

Table 5 showed results on multinomial regressions. Multinomial logistic regression analysis showed that aging, being female, being a rural resident, being widowed/divorced/unmarried, having a primary school and lower education, experiencing inconvenient reimbursement of medical expenses, facing financial difficulties, and living in northern China were positively correlated with frailty and pre-frailty in older adults.

**Table 5:** Related factors associated with frailty and pre-frailty of older adults by multinomial logistic regression.

Variables		Pre-frailty vs. robustness	Frailty vs. robustness		
		OR (95% CI)	*p*-value	OR (95% CI)	*p*-value
Sex	Male	1 (ref)			
	Female	1.276 (1.250–1.302)	<0.0001	1.495 (1.442–1.549)	<0.0001
Age (years)	60–64	1 (ref)			
	65–69	1.299 (1.266–1.333)	<0.0001	1.607 (1.524–1.694)	<0.0001
	70–74	1.649 (1.601–1.698)	<0.0001	2.511 (2.376–2.654)	<0.0001
	75–79	1.936 (1.872–2.001)	<0.0001	3.826 (3.614–4.051)	<0.0001
	80–84	2.270 (2.178–2.365)	<0.0001	5.698 (5.348–6.070)	<0.0001
	≥85	2.618 (2.480–2.764)	<0.0001	9.473 (8.800–10.198)	<0.0001
Urban or rural area	Urban	1 (ref)			
	Rural	1.150 (1.126–1.174)	<0.0001	1.231 (1.188–1.276)	<0.0001
Marital status	Married	1 (ref)			
	Widowed/divorced/unmarried	1.068 (1.038–1.099)	<0.001	1.231 (1.176–1.289)	<0.0001
Education	Primary school or lower	1 (ref)			
	Middle school	0.876 (0.853–0.900)	<0.0001	0.753 (0.715–0.793)	<0.001
	High school or higher	0.856 (0.827–0.886)	<0.0001	0.655 (0.611–0.702)	<0.001
Ethnicity	Han	1 (ref)			
	Non-Han	1.052 (1.009–1.097)	0.017	0.969 (0.903–1.040)	0.3797
Living alone	No	1 (ref)			
	Yes	2.405 (2.316–2.497)	<0.0001	2.361 (2.237–2.493)	<0.0001
Economic status	Rich	1 (ref)			
	Adequate	1.387 (1.350–1.426)	<0.0001	1.586 (1.502–1.674)	<0.0001
	Poor	2.512 (2.433–2.595)	<0.0001	4.319 (4.075–4.578)	<0.0001
Medicare	Yes	1 (ref)			
	No	0.897 (0.785–1.025)	0.1099	0.886 (0.711–1.106)	0.2848
Medical cost reimbursement	Convenient	1 (ref)			
	Less convenient	1.028 (1.002–1.054)	0.0328	0.990 (0.948–1.034)	0.6489
	Inconvenient	1.258 (1.204–1.313)	<0.0001	1.509 (1.412–1.612)	<0.0001
Southern or northern	Southern	1 (ref)			
	Northern	1.394 (1.362–1.427)	<0.0001	2.175 (2.095–2.257)	<0.0001

CI, confidence interval; OR, odds ratio.

## Discussion

Our cross-sectional study, which included the largest sample size to date in China and covered 31 provinces/municipalities/autonomous regions, used a rigorous sampling design and quality control to accurately report the prevalence of frailty and pre-frailty among older adults in China based on “self-reported data” analysis. Our findings suggested that the overall age-sex standardized prevalence of frailty and pre-frailty is 9.5% (95% CI 9.4–9.7) and 46.1% (45.9–46.3), respectively. This represents 25.15 million and 121.64 million people aged 60 years or older in China in 2020, and our study reported a higher prevalence of frailty than two previous large studies. The China Health and Retirement Longitudinal Study (CHARLS) (5,301 participants) conducted in 2011–2012 reported a 7.0% prevalence of frailty in people aged 60 years or older, assessed using the physical frailty phenotype (19), the Kadoorie Biobank study (74,820 participants) conducted from 2004 to 2008 reported a prevalence of 8.9% in older adults aged 65 years or older, assessed using the FI (20). Our findings are generally consistent with the 9.9% prevalence of frailty in people aged 60 years or older assessed using the FI as reported in the China Comprehensive Assessment Study on Geriatrics (CCGAS) conducted in 2011–2012 (21). We reported a relatively lower prevalence of frailty in older adults compared with other countries. In a recently published article compiling 240 studies from 62 countries and territories, the prevalence of pooled frailty as assessed by the physical frailty phenotype was 12%, while the prevalence as assessed by the FI was 24%, for pre-frailty, this was 46% and 49%, respectively (22). A cross-national study on the prevalence of frailty assessed using the physical frailty phenotype in older adults conducted in low-or middle-income countries showed that the prevalence was low in rural (5.4%) and urban (9.1%) China and varied between 12.6% and 21.5% in other sites (23). Global differences in the prevalence of frailty in older people may be due to different economic medical conditions, environmental risks and genetic factors. In addition, heterogeneity in study methodology, including the use of different diagnostic criteria, could influence the results. Studies have shown the lowest incidence of frailty detected when using physical frailty phenotype, while the FI produces higher estimates (8, 22). This may be related to conceptual differences between the two measures, with the FI representing the accumulation of health deficits over time, whereas Fried’s frailty phenotype is a clearer distinction between signs and symptoms of frailty and disability (1, 12). Therefore, there is an urgent need to improve the accuracy of prevalence estimates in older adults using a uniform frailty assessment methodology.

Our study addressed several gaps in previous studies on frailty in older adults in China. Firstly, we reported the prevalence of frailty and pre-frailty in older adults in 31 provinces/municipalities/autonomous regions in mainland China. Secondly, we reported the prevalence of frailty and pre-frailty in older adults from ethnic minorities in China. Finally, we identified for the first time the association between the payment ratio of medical reimbursement and frailty.

As the prevalence of frailty and pre-frailty increases dramatically with age, the reported geographic differences may be traced to different age structures. Therefore, we calculated the weighted prevalence of frailty and pre-frailty among the older adults in 31 provinces/municipalities/autonomous regions in mainland China using the 2020 census data. This comparison is more meaningful for identifying the underlying causes of regional differences in the prevalence of frailty and pre-frailty in older adults and for designing targeted public health interventions. Our study revealed significant geographic differences in the weighted prevalence of frailty and pre-frailty among the older adults among provinces/municipalities/autonomous regions in mainland China. The prevalence of pre-frailty and frailty in the older adults in northern China was higher than that in southern China. The northwest region in northern China had the highest prevalence of frailty, followed by north and northeast China, and the southeastern and southern regions of China have the lowest prevalence. Unlike our study, CHARLS found that the prevalence of frailty was lowest in the northeast (19). However, the relatively small number of frail older adults in different administrative regions in the CHARLS study may have contributed to the uncertainty and underrepresentation of the findings.

Our study identified several facorts associated with frailty and pre-frailty among older adults in China, including increasing age, female sex, rural residence, poor marital status, fewer years of education, living alone, and difficult economic status and living in northern China, these findings are consistent with other previously published studies (15, 19, 21, 24). Studies have shown that ageing is an independent risk factor for the development of frailty, as degenerative changes in several physiological systems lead to decreased function and increased risk of frailty in older adults as they age (3, 25). Older women with decreased levels of oestrogen may be at higher risk of frailty due to decreased muscle strength (26). access to education and access to health care are important markers of socio-economic status (27), and difficulties in accessing health care due to difficult economic status may lead to an increased risk of frailty (28, 29), and poor marital status and living alone can also increase the risk of frailty by experiencing loneliness and depression (30). Research shows that frailty and chronic disease and disability overlap, that frailty is an intermediate state between chronic disease and disability, that it is the beginning of a vicious cycle of geriatric syndromes, and studies show that multimorbidity is a risk factor for frailty (19, 31). We found that the rates of low educational attainment, living alone, Self-reported multimorbidity and disability were higher among female older adults compared with their male counterparts; the rates of low educational attainment, living alone, difficult financial situation, poor access to medical reimbursement, Self-reported multimorbidity and disability were higher among rural older adults compared with urban older adults; and the rates of difficult financial situation, poor access to medical reimbursement, Self-reported multimorbidity and disability were higher among older adults in northern China compared with that in southern China. These demographic differences and factors associated with frailty may partially explain the differences in the prevalence of frailty among older adults by gender, rural and urban areas, and in northern and southern China. These findings from our study inform policy makers to take targeted measures to reduce and prevent frailty, particularly among women, rural residents and older adults living in northern China.

Our study found that the availability of health insurance was not a siginificant factor associated with frailty among older adults in China, this may be due to the increased health insurance coverage in China in recent years, with approximately 99% of older adults having health insurance. However, our study found that difficulty in reimbursing medical expenses was a relevant factor for frailty, hightlighting the importance of increasing the payment ratio of medical expense reimbursement.

Our study found no difference in the prevalence of frailty between ethnic minority and Han Chinese older adults in China, but the prevalence of pre-frailty was higher among ethnic minority older people than that of Han Chinese. Moreover, a higher proportion of ethnic minority older people had low educational attainment, difficult financial status and inconvenient medical reimbursement compared to Han Chinese. Therefore, improving access to education, eliminating economic disparities and increasing the rate of medical reimbursement for ethnic minorities could help prevent the risk of frailty and pre-frailty among ethnic minority older people.

The current prevalence of frailty in China that we reported does not reflect global prevalence trends. Although the prevalence of frailty in the Chinese population is relatively low compared to global trends, our study found that the prevalence of pre-frailty is similar to global levels (22). Pre-frailty is the state between robustness and frailty and is generally considered to be a clinically asymptomatic stage (32). Studies have shown that older adults with pre-frailty have a higher risk of adverse clinical outcomes and an increased risk of progression to frailty (33–37). The prevalence of frailty will increase as many pre-frail people progress to frailty in the coming years, and as China entered a phase of rapid population ageing, the older population size and the risk factors such as chronic non-communicable diseases increase (38, 39), preventive management of frailty should be a top priority for health policymakers. Our study found that the relevant factors for pre-frailty and frailty are similar; therefore, the whole chain of risk factors, pre-frailty and frailty needs to be managed effectively, and early identification of pre-frail people and effective intervention is an important means of delaying and reducing the incidence of frailty in older people. However, unlike frailty, there is less theoretical evidence for pre-frailty, and pre-frailty is a concept that needs further research in the context of population ageing (32).

Based on the prevalence of frailty and pre-frailty in older people and their associated risk factors, frailty poses significant impacts on the healthcare system and society as a whole. Frailty can lead to increased hospitalization, prolonged hospital stay, and increased medical costs, which can burden the healthcare system. The economic consequences of frailty are also severe, as frail older adults are more likely to require long-term care, which can be expensive and lead to economic pressure on families and the healthcare system. Furthermore, frailty can lead to decreased productivity and increased absenteeism among caregivers, which can affect the economy. Socially, frailty can lead to social isolation and decreased quality of life among older adults, which can have a ripple effect on families and communities. To effectively control and manage frailty, we recommend establishing a comprehensive public health system in China. This includes increasing healthcare professionals’ awareness of frailty in older adults, strengthening public health education to improve public awareness of frailty, pre-frailty, and related risk factors, establishing a national monitoring network for frailty and pre-frailty, and regularly conducting assessments. By identifying people at risk of frailty and providing appropriate interventions, we can reduce medical costs, improve the quality of life of older adults, and reduce the burden on caregivers and the healthcare system. Intervention measures can include exercise programs, nutrition education, and medication management. In addition, social support programs and caregiver support can help reduce social isolation and improve quality of life. By investing in interventions for vulnerable populations, we can improve the health and well-being of older adults and reduce the economic and social consequences of frailty.

Our study has several limitations. First, while the minimum number of items used to construct the FI was 28, which is lower than in some other studies (mostly at 30 or more), the accuracy of the 28-item construction of the FI has been confirmed in a previous study (20). Second, the items used to construct the FI lacked deficits related to cognitive ability, which is an important component of geriatric syndrome. Third, our study cannot exclude potential recall bias, for example in the diagnosis of chronic diseases. Fourth, due to the cross-sectional nature of the study, it is difficult to establish a causal relationship between the factors of interest and frailty. Fifth, our study used the FI to assess frailty and therefore did not analyse specific chronic diseases and specific health conditions associated with frailty and pre-frailty. Analysing specific chronic conditions such as diabetes, heart disease and chronic obstructive pulmonary disease associated with frailty and pre-frailty would provide a better understanding of the complex interactions between chronic conditions and frailty. Analysing of specific health conditions such as cognitive impairment and falls, where increased risk of cognitive impairment and falls is associated with reduced physical functioning, may help to understand the development of frailty. One potential future direction for frailty research in older adults is longitudinal studies that assess the extent of frailty over time. This could help us to better understand the progression of frailty and identify potential risk factors that contribute to its development. In addition, qualitative research exploring the lived experiences of frail people could help us gain a deeper understanding of the impact of frailty on individuals and their families. Intervention studies that assess the effectiveness of specific interventions in reducing frailty are also important. This can help us to identify the most effective interventions to prevent or reduce frailty and provide evidence-based recommendations for healthcare providers and carers. Another potential area of research is the development of new technologies and interventions to help older adults maintain their physical and cognitive function and prevent frailty. For example, wearable technology and telehealth interventions could help older adults monitor their health and receive timely interventions when necessary.

## Conclusion

This study, the largest representative survey of frailty in older adults in China to date, suggests that frailty and pre-frailty are highly prevalent among older adults in China, particularly in northern China, and there are common relevant factors. Therefore, to prevent and treat frailty in older people, targeted measures are needed, including increased investment in healthcare in underdeveloped areas, better reimbursement of healthcare costs, and improved management of frailty-relevant factors such as chronic diseases and strengthening health education for the older adults such as exercise, diet, supplementation of vitamins or minerals that are lacking, exposure to sunlight, etc. By promoting frailty screening and optimizing frailty management in older adults, it can help to reduce the burden of frailty among older adults in China.

## Data availability statement

The original contributions presented in the study are included in the article/supplementary material, further inquiries can be directed to the corresponding author.

## Ethics statement

The studies involving human participants were reviewed and approved. The study protocol was approved by National Bureau of Statistics [No. (2014) 87] and the ethics committee of Beijing Hospital (2021BJYYEC-294-01). The patients/participants provided their written informed consent to participate in this study.

## Author contributions

X-zZ, Y-yL, and NJ wrote the various drafts of the manuscript. X-zZ, L-bM, and JS conducted the statistical analyses. CZ, XH, J-bH, J-yL, D-sW, HL, HW, and XQ were involved in data interpretation. D-pL, Q-xZ, JL, and X-zZ conceived and designed this study. Drafts of the manuscript were revised for important scientific content by X-zZ, L-bM, Y-yL, NJ, JS, CZ, XH, J-bH, J-yL, D-sW, HL, HW, XQ, Q-xZ, JL, and D-pL. D-pL is the guarantor of this work and, as such, had full access to all the data in the study and takes responsibility for the integrity of the data and the accuracy of the data analysis. All authors contributed to the article and approved the submitted version.

## Funding

The present study was funded by the National Key R&D Program of China (Grant nos. 2020YFC2003000 and 2020YFC2003001). The study sponsors were not involved in the design of the study, the collection, analysis, and interpretation of data, writing the report, or the decision to submit the report for publication.

## Conflict of interest

The authors declare that the research was conducted in the absence of any commercial or financial relationships that could be construed as a potential conflict of interest.
